# Trends in Extended Spectrum Beta-Lactamase (ESBL) Producing Enterobacteriaceae and ESBL Genes in a Dutch Teaching Hospital, Measured in 5 Yearly Point Prevalence Surveys (2010-2014)

**DOI:** 10.1371/journal.pone.0141765

**Published:** 2015-11-03

**Authors:** Ina Willemsen, Stijn Oome, Carlo Verhulst, Annika Pettersson, Kees Verduin, Jan Kluytmans

**Affiliations:** 1 Laboratory for Microbiology and Infection Control, Amphia Hospital, Breda, The Netherlands; 2 Department of Medical Microbiology and Infection Control, VU University Medical Center, Amsterdam, The Netherlands; 3 Julius Center for Health Sciences and Primary Care, UMC Utrecht, Utrecht, the Netherlands; University of Cambridge, UNITED KINGDOM

## Abstract

This paper describes the trends in prevalence of ESBL producing Enterobacteriaceae (ESBL-E) and ESBL genes, measured in five consecutive yearly Point Prevalence Surveys (PPS). All patients present in the hospital and in a day-care clinic (including patients on dialysis) on the day of the survey, were screened for perianal ESBL-E carriage. Perianal swabs were taken and cultured using an enrichment broth and a selective agar plate. Both phenotypic and genotypic methods were used to detect the production of ESBL, presence of ESBL-genes and clonal relatedness. Out of 2,695 patients, 135 (5.0%) were tested ESBL-E positive. The overall ESBL-E prevalence was stable over the years. Overall 5.2% of all ESBL-E were acquired by nosocomial transmission. A relative decrease of CTX-M-1-1-like ESBL genes (from 44 to 25%, p = 0.026) was observed, possibly related to the strong (>60%) decrease in antibiotic use in livestock in our country during the same period.

## Introduction

Resistance to β-lactams in Gram-negative bacteria is rapidly increasing and is mainly related to the dissemination of mobile genetic elements encoding β-lactamases. Extended spectrum β-lactamases (ESBL) are found relatively frequent in Dutch hospitals [1–3]. Beside antibiotic stewardship, high quality infection control is considered to be the most important strategy to fight resistance [4]. To judge the quality of the infection control policy, we depend on information about the local endemic level and epidemiology of specific resistant microorganisms and of resistance genes. Information about resistance can be obtained from clinical cultures but is affected by the indications for sampling and is not representative of the reservoir in the gut [3]. Therefore, the reservoir of asymptomatic carriage may be missed when relying solely on clinical cultures [5]. A prevalence survey based on perianal swabs can be performed to obtain accurate insight in the epidemiology of ESBL-producing Enterobacteriaceae (ESBL-E) in a healthcare institute. Since 2010, five yearly Point Prevalence Surveys (PPS) have been performed in a large Dutch teaching hospital. This paper describes the trends in Extended Spectrum Beta-Lactamase producing Enterobacteriaceae (ESBL-E) and ESBL genes over time.

## Methods & Material

Five yearly PPS were performed from 2010 through 2014, in the month of November. All hospitalised patients, including patients on dialysis and day-care, were screened for ESBL-E carriage using perianal swabs (Eswab, Copan, Italy). After vortexing, the swab was plated on Blood Agar plate (growth control, performed since 2011) and the liquid Amies eluent was inoculated in selective tryptic soy broth, containing cefotaxime (0.25 mg/L) and vancomycin (8 mg/L) (TSB-VC). After 18–24 hours of incubation (35–37°C), 10 μl TSB-VC was sub-cultured on both sides of an EbSA agar plate (AlphaOmega, 's-Gravenhage, Netherlands). The Extended Beta-Lactamase Screening Agar (EbSA) plate consists of a split MacConkey agar plate containing ceftazidime (1.0 mg/L) on one side and cefotaxime (1.0 mg/L) on the other side. Both sides contain cloxacillin (400 mg/L) and vancomycin (64 mg/L) for inhibition of AmpC beta-lactamase-producing bacteria and Gram-positive bacteria, respectively. Subsequently the plates were incubated aerobically at 35 to 37°C for 18 to 24 hours. Species identification and susceptibility testing was performed for all oxidase negative isolates that grew on either side of the agar, by VITEK2 GN (between 2010 and 2013) or MALDI-TOF (bioMérieux, Marcy l’Etoile, France) (in 2014), and VITEK 2 AST N199 (bioMérieux, Marcy l’Etoile, France) respectively.

The presence of ESBL in isolates with a MIC of > 1 mg/L for ceftazidime and/or cefotaxime was phenotypically confirmed with the combination disk diffusion method for cefotaxime, ceftazidime, and cefepime with and without clavulanic acid (Rosco, Taastrup, Denmark). Test results were considered positive if the inhibition zone around the disk with clavulanic acid was increased by 5 mm for the combination [6]. Identification of the ESBL genes was performed using the Check-MDR CT103 microarray (Check-Points, Wageningen, The Netherlands) [7]. This assay identifies the β-lactamase genes of TEM, SHV and CTX-M, and is able to detect single nucleotide polymorphisms in TEM en SHV genes, thus discriminating between ESBL and non-ESBL TEM and SHV variants.

Isolates containing the same ESBL gene from patients that were admitted on the same ward were selected for Amplified Fragment Length Polymorphism (AFLP) testing to identify clonal clusters. If one patient contained more than one ESBL positive strain from different genus or species, or with different resistance genes, all strains were included.

AFLP typing was performed as described by Mohammadi et al. [8]. Restriction was performed with EcoR1 and MseI. After adapter ligation, primers EcoA (FAM-labeled) and MseC were used for PCR. DNA fragments were separated on an ABI PRISM 3130 sequencer (Applied Biosystems). Data were analysed with Genescan analysis software (Applied Biosystems) and BioNumerics software package, version 6.6 (Applied Maths, Sint Martens Latem, Belgium). Similarity coefficients were calculated with Pearson correlation and dendrograms were obtained by the unweighted pair group method using arithmetic averages (UPGMA) clustering. The analysis was performed for fragments with lengths between 60 and 600 bp. Genetic relatedness was determined on basis of both visual and computerised interpretation of AFLP patterns. The person who did the interpretation was not aware of the epidemiological information of the patients (observer blind analysis).

Research was conducted to detect nosocomial transmission, excluding patients admitted to the day-care and dialyses unit. Nosocomial transmission was considered to have occurred if genotypically identical strains (based on AFLP) were detected in two or more patients admitted on the same ward during one PPS [9]. Horizontal gene transfer between different species or strains of the same species was not investigated. Per PPS the prevalence of ESBL-E carriage was calculated and if applicable corrected for clonal transmission (with the assumption that one case per cluster was the index case).

The yearly PPS for ESBL-E carriage was part of the infection control hospital policy and is approved by the management of the hospital. Participation was based on a treatment contract that the hospital has with all admitted patients. This includes the participation with all non-invasive procedures that are part of the hospital’s patient safety and infection control program. The ESBL-E screening was part of that program. If patient indicated that they did not want to take part in the screening they were excluded (opt-out).

### Data collection and statistical analysis

Patient samples were taken by nursing staff or by patients themselves whatever the patient preferred. The Infection Control Practitioner (ICP) collected the information on the patient characteristics.

According to the Dutch regulation for research with human subjects, neither medical nor ethical approval, was required to conduct the surveillance since it was part of the local hospital policy, all data were processed anonymously. Data were analysed with Statistical Package for Social Science software (SPSS Version 19). A trend analyses in the prevalence of ESBL genes was performed in a regression analysis using a logarithmic function. The Pearson correction coefficient was calculated to determine the correlation between length of stay in the hospital and ESBL-E carriage and type of ESBL genes. Statistical significance was accepted at *p* <.05.

## Results

A total of 3160 patients were eligible for ESBL-E screening. Of those, 2724 patients were screened, which results in a response rate of 86% (Table 1). Of those, 2695 cultures (85.3%) were evaluable (positive growth control on blood agar). The median age of the screened patients was 66 years (range 0–99), and about half of the patients were female. Tables 2 and 3 shows the distribution of patients across various medical specialties and the prevalence of ESBL within the medical specialties. Overall, 84% (N = 2252) of the evaluable patients were admitted to a clinical ward within the hospital, with a median length of stay of 3 days (range 0–90 days), the other 16% (N = 443) were day-care patients (including dialysis).

**Table 1:** ESBL prevalence over time, including bacterial species and ESBL genes.

	2010	2011	2012	2013	2014	Overall
Hospitalised patients (incl. day-care), No.	667	642	598	601	652	3160
No perianal swab taken, No.	108	72	88	85	83	436
Negative growth control, No.	n.a.	6	3	8	12	29
Evaluable cultures (patients), No.	559	564	507	508	557	2695
prevalence ESBL-E carriage						
ESBL positive patients, No. (%) ^&^	25 (4.5%)	27 (4.8%)	20 (3.9%)	26 (5.1%)	37 (6.6%)	135 (5.0%)
Primary, No.^#^	25	26	15	26	36	128
Secondary, No. ^$^	0	1	5	0	1	7
ESBL producing species, No.						
Total ESBL isolates, No.	25	30	22	30	38	145
Unique & primary ESBL isolates, No.	25	29	16	30	37	137
*Escherichia coli*, No.	24	24	10	25	29	112
*Klebsiella pneumonia*, No.	1	1		4	3	9
*Klebsiella oxytoca*, No.				1		1
*Pantoea agglomerans*, No.		1				1
*Enterobacter cloacae*, No.		1	3		3	7
*Enterobacter aerogenes*, No.			1			1
*Citrobacter freundii*, No.		1	1		1	3
*Morganella morganii*, *No*		1	1		1	3
Secondary ESBL isolates, No	0	1	6	0	1	8
*Escherichia coli*, No.		1	3			4
*Enterobacter cloacae*, No.			3			3
*Klebsiella pneumonia*, No.					1	1
ESBL genes (excl. secondary cases)						
CTX-M-1 group, No.	17	22	9	19	26	93
*CTX-M-1 like*, No.	11	10	4	5	10	40
*CTX-M-15 like*, No.	4	10	3	7	16	40
*Other*, No.	2	2	2	7	0	13
CTX-M-9 group, No.	5	3	5	9	10	32
SHV-SNP, No.	3	3	3	1	4	14
TEM-SNP, No.	0	1	1	1	0	3

^&^ = Number of ESBL positive patients divided by the number of evaluable cultures (patients)

^#^ = Primary = patients with a unique isolates and index patients

^$^ = Secondary = patients with ESBL isolates caused by nosocomial transmission

**Table 2:** Baseline characteristics of screened patients.

	2010	2011	2012	2013	2014
Total patients with evaluable cultures	559	564	507	508	557
Female, No. (%)^#^	285 (51%)	271 (48%)	244 (48%)	249 (49%)	289 (52%)
age, median, No. (range)	67 (0–95)	64 (0–99)	67 (0–97)	65 (0–93)	65 (0–99)
Hospitalisation > 2 days, No.(%)	275 (49.2%)	273 (48.4%)	265 (52.3%)	231 (45.4%)	271 (49.5%)
Length of stay, median, No. days (range)	2 (0–90)	2 (0–48)	2 (0–79)	2 (0–51)	2 (0–70)
Patients in day-care, No. (%)^#^	113 (20%)	99 (18%)	64 (13%)	66 (13%)	90 (17%)
medical specialty, No. (%)^#^					
anesthesiologie (non-ICU),	17 (3%)	15 (3%)	9 (2%)	10 (2%)	9 (2%)
cardiology	59 (11%)	39 (7%)	53 (11%)	46 (9%)	50 (9%)
geriatrics	7 (1%)	13 (2%)	10 (2%)	11 (2%)	11 (2%)
Intensive Care Unit (ICU)	15 (3%)	17 (3%)	14 (3%)	17 (3%)	14 (3%)
internal medicine^#^	128 (23%)	137 (24%)	130 (26%)	132 (26%)	134 (24%)
neurology	43 (8%)	32 (6%)	29 (6%)	31 (6%)	35 (6%)
obstetrics and gyneacology	27 (5%)	48 (9%)	25 (5%)	43 (9%)	21 (4%)
orthopedic surgery	58 (10%)	38 (7%)	29 (6%)	34 (7%)	42 (8%)
otorhinolaryngology	10 (2%)	12 (2%)	9 (2%)	8 (2%)	20 (4%)
pediatrics	23 (4%)	28 (5%)	42 (8%)	24 (5%)	31 (6%)
pulmonary diseases	38 (7%)	42 (8%)	41 (8%)	32 (6%)	41 (7%)
surgery, cardiothoracic	21 (4%)	24 (4%)	15 (3%)	17 (3%)	17 (3%)
surgery, general	82 (15%)	86 (15%)	76 (15%)	86 (17%)	94 (17%)
urology	13 (2%)	20 (4%)	19 (4%)	17 (3%)	21 (4%)
other specialty	19 (3%)	12 (2%)	6 (1%)	0	13 (3%)

^#^ = percentages refer to the number divided by the total patients with evaluable cultures

**Table 3:** ESBL-E prevalence within the different medical specialties.

medical specialty	N	ESBL-E positive, No. (%)^#^
anesthesiologie (non-ICU)	60	4 (6%)
cardiology	247	14 (5.7%)
geriatrics	94	7 (7,4%)
Intensive Care Unit (ICU)	424	19 (4,5%)
internal medicine^#^	661	29 (4,4%)
neurology	59	1 (1,7%)
obstetrics and gyneacology	148	9 (6,1%)
orthopedic surgery	194	9 (4.6%)
otorhinolaryngology	170	9 (5.3%)
pediatrics	164	3 (1,8%)
pulmonary diseases	201	12 (6.0%)
surgery, cardiothoracic	90	6 (6,7%)
surgery, general	52	6 (11,5%)
urology	54	4 (7,4%)
other specialty	77	3 (3,9%)
overall	2695	135 (5.0%)

^#^ = percentages refer to the number of ESBL-E positive patients divided by the total within medical specialty

**Fig 1:**
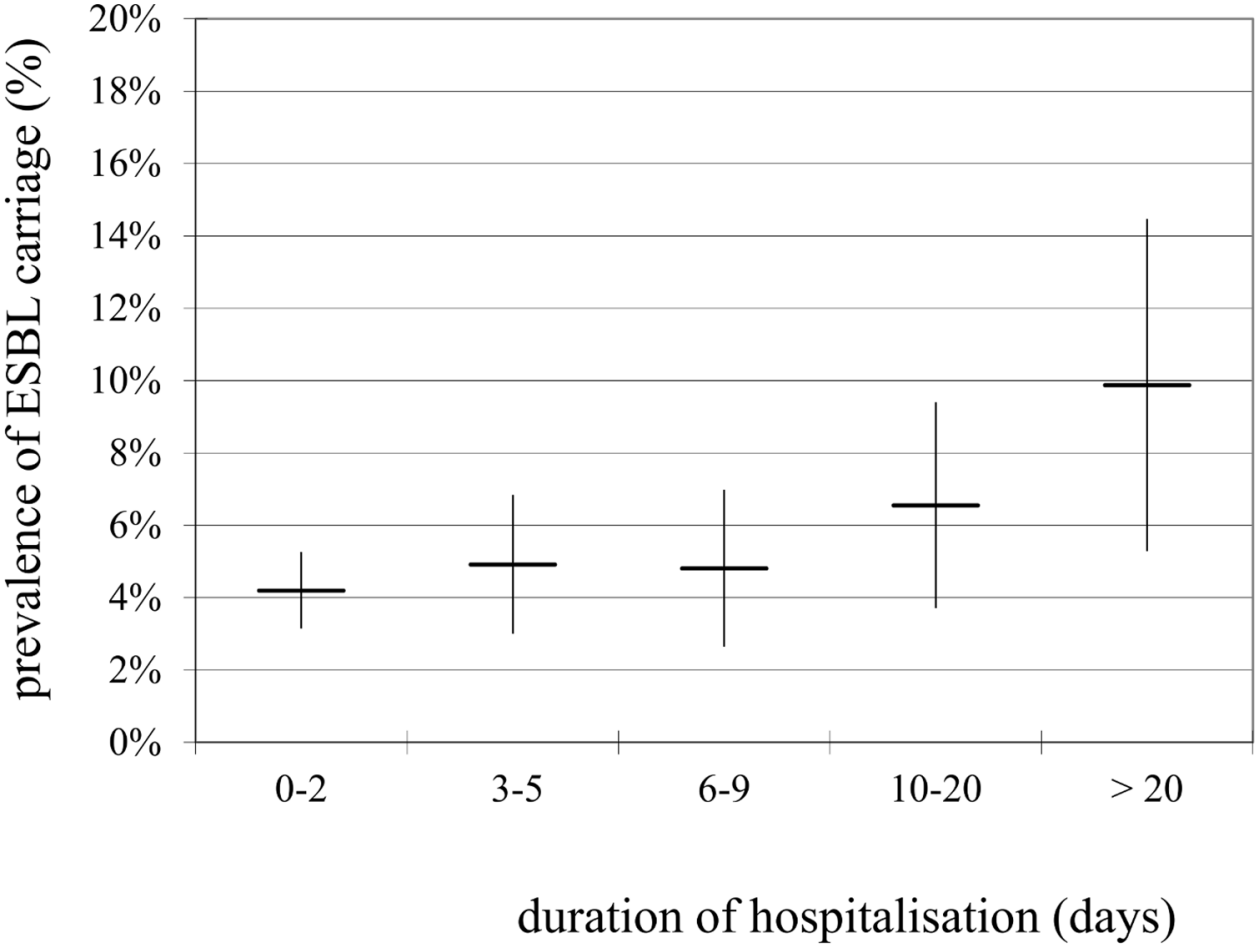
ESBL-E prevalence in relation with the duration of hospitalisation (patients in day-care were excluded). Vertical bars represent the 95% confidence intervals.

The yearly ESBL-E prevalence, from 2010 to 2014, was stable with an overall prevalence of 5.0% (N = 135), shown in Table 1. The prevalence in hospitalised patients with an admission time of 2 days or less was not significantly different from patients with an admission time longer than 2 days, 58/1381 (4.2%) and 77/1314 (5.9%), respectively (RR = 1.2, 95% CI = 0.99–1.50). However, when hospitalisation was prolonged, relatively more patients were detected as ESBL-E positive (p = 0.003 trend analysis). Fig 1 shows the observed ESBL-E prevalence in relation to the duration of hospitalisation (excluding day-care and dialyses).

From the 135 ESBL positive patients, 145 ESBL-E were cultured. *E*. *coli* was the predominant species, (n = 112, 77%) followed by *K*. *pneumoniae* (n = 9, 6%) and *E*. *cloacae complex* (n = 7, 5%) as shown in Table 2. A total of 152 ESBL genes were detected. ESBL genes belonging to the CTX-M-1 group were most frequently found (n = 95, 63%). Furthermore, 30 ESBL genes from the CTX-M-9 group, fourteen SHV genes and three TEM genes were detected. No relation was found between type of ESBL gene and the length of stay in the hospital.

**Fig 2:**
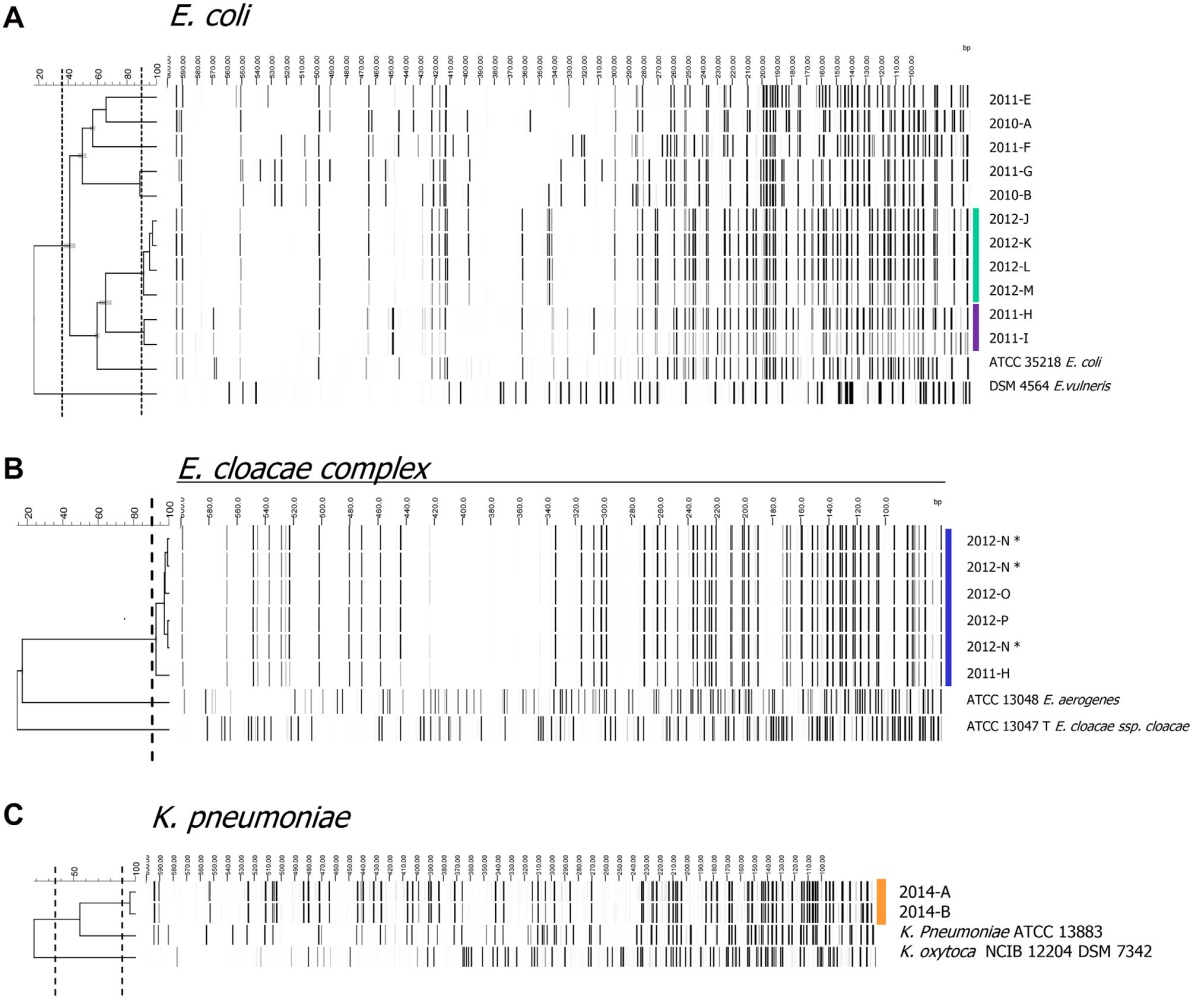
AFLP patterns from all ESBL-E *E*. *coli*, *E*. *cloacae complex* and *K*. *pneumonia* eligible for cluster analyses. Strains clustering with a similarity between 90 and 100% were defined as identical strains. Strains clustering with a similarity above 35% were defined as different strains of the same species and strains clustering with a similarity below 35% were defined as different species. Identical strains are indicated in color. Each strain was coded with the number of the year in combination with a letter. * three ESBL positive strains cultured in a sample from patient 2012-N showed fenotypic differences. Therefore AFLP analyses was performed from all three samples.

Overall 5.2% of all ESBL-E were considered to be acquired by nosocomial transmission. Fig 2 shows the AFLP patterns of genotypically identical ESBL-positive *E*. *coli*, *E*. *cloacae complex* and *K*. *pneumoniae* that were considered as nosocomial clusters (same species, same ESBL gene, hospitalisation on the same ward). In 2011, one case of nosocomial transmission of a *bla*_CTX-M1-1_ producing *E*. *coli* was observed in the internal medicine ward. In 2012, 2 clusters were found; 4 identical CTX-M9 group producing *E*. *cloacae complex* strains in geriatrics, and 4 identical CTX-M9 group producing *E*. *coli* strains in neonatology (including a triplet). In 2014, one case of transmission with a *bla*_CTX-M1-15_ producing *K*. *pneumoniae* was detected in neonatology. All clusters involved one or more patients with a length of stay in the hospital of more than 7 days.

**Fig 3:**
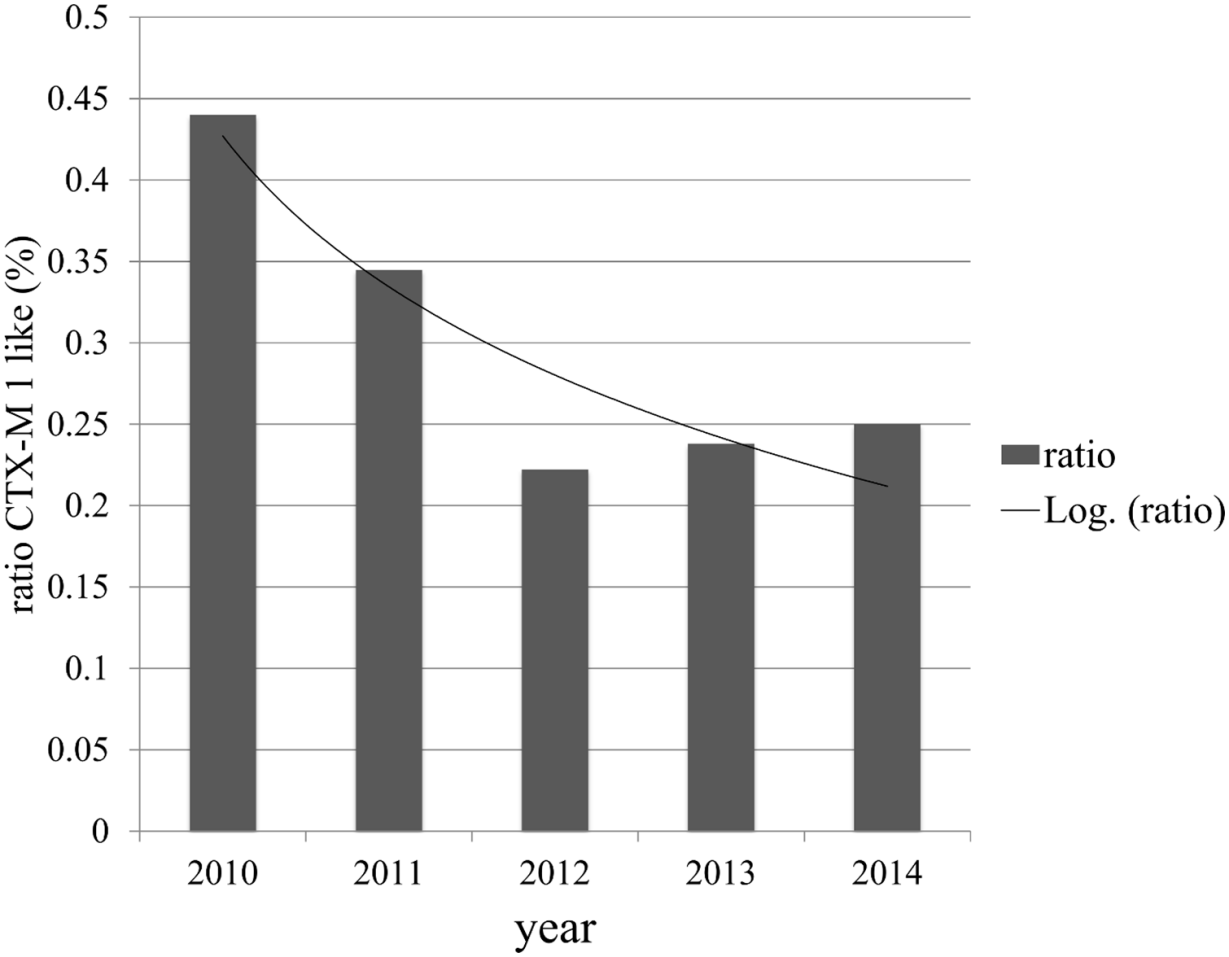
Proportion of CTX-M-1 like ESBL genes over time. The vertical bars represent the percentage of CTX-M-1 like ESBL genes divided by the total number of ESBL genes. The line represents the logarithmic trendline.

After excluding the ESBLs acquired by nosocomial transmission, trend analyses of the ESBL genes showed a significant decrease of the proportion of CTX-M-1 like ESBL over the years (p = 0.026, Fig 3).

## Discussion

This paper describes a prevalence of ESBL-E carriage of 5%, measured in five yearly point prevalence surveys. The prevalence was stable over time and transmission was rarely observed. Only 5.2% of all ESBL-E were considered acquired by nosocomial transmission.

The surveillance was performed in a non-outbreak situation and the surveillance included all hospitalised patients (including day-care and dialysis). This gives us useful information about the local epidemiology in our hospital, but makes it difficult to compare with prior published data. Most publications describe outbreak situations or specific patients populations, such as travellers or patients with gastrointestinal complaints. For example, in our national capital Amsterdam, a ESBL-E carriage rate of 10.1% was detected [10]. This surveillance was performed in a population of community patients with gastrointestinal complaints. The prevalence in asymptomatic, healthy, community dwelling individuals without a recent travel history is not known.

Among day-care patients and patients in their first two days of hospitalisation the prevalence was already more than 4%. This indicates that ESBL-E is also common in the Dutch community, nowadays. However, some of these patients may have had a recent hospitalisation or can be frequent visitors of healthcare settings. This information is not available.

When hospitalisation was prolonged, relatively more ESBL-E was detected, while transmission was rarely observed. There are multiple possible explanations for this observation. It could be caused by antimicrobial selection of already present, but undetected, ESBL-Es (low bacteria load). Alternatively, nosocomial transmission did occur but was not detected because the index-patient had already been discharged. Possibly, ESBL-E carriers have a longer length of stay in the hospital due to an ESBL-E infection or due to comorbidity. One can only speculate about the relation between increased detection of ESBL-E carriage and the length of stay in the hospital, based on our surveillance. More in depth studies are needed to provide scientific explanations.

The ESBL screening in this report was not primarily performed to detect nosocomial transmission. Nevertheless, the presence of an on-going outbreak would have been detected, especially in our setting with a relatively low prevalence of antimicrobial resistance. In 2011, 2012 and in 2014 a few cases of nosocomial transmission were detected. Transmission could have occurred before admission, e.g. in a nursing home, or could have been caused by another common source outside the hospital. However, we found a clear epidemiological link within the ward (e.g. room or patient) in all strains with a similar typing result. Furthermore, none of these patients lived in the same nursing home before admission. The reported cases of nosocomial transmission were reported to the ward and the screening was repeated. No new ESBL positive strains were found.

This study focussed on clonal dissemination of ESBL-E and the role of mobile genetic elements was not investigated. However, we know that multidrug resistance is often associated with the spread of transmissible plasmids and integrons, and may have a large contribution to the spread of resistance within the hospital setting [11].

In a nursing home, nearby our hospital, a large outbreak of ST131 with CTX-M-15 like positive *E*. *coli* was detected after a prevalence survey for ESBL-E carriage [12]. The differences in ESBL prevalence between our hospital and the nearby nursing home (ESBL prevalence of 20.6%) is remarkable, because frequent transfer from patients between these care facilities occurred [12]. A possible explanation could be the difference in duration of stay in the nursing home. The average length of stay in our hospital is 5.7 days while residents in the nursing home stay there on average for more than one year [13]. We have no indications that patients with dementia more often refused participation to the ESBL-E screening. For example the department of geriatrics had a participation rate that was similar to other departments. The participation rate was lower in day-care (p<0.001) and for relatively younger patients (mean age of not cultured patients was 54 years against 60 years in the group of cultured patients, p<0.001).

A limitation of this PPS is that nosocomial acquisition of ESBL-E may be undetected due to discharge of the patients before the day of the PPS. Furthermore, the probability to detect ESBL-E acquisition will be lower in the first days after acquisition. The minimal time period from ESBL-E acquisition to the presence of detectable amounts of ESBL-E in faecal samples is currently unknown.

Patients were allowed to sample themselves if they wanted; this could have influenced the quality of sampling and must be regarded as a limitation. Since 2011 we always directly plated a Blood Agar plate to control for the quality of sampling, and check if sampling took place anyway. The number of negative growth controls (Table 1) was very low. We therefore consider the data sufficiently reliable.

In our surveillance, the most common ESBL found in perianal swabs in 2010 and 2011 was CTX-M-1 like ESBL. Research performed in the same period showed that CTX-M-1 like ESBL was also the most frequently observed ESBL gene in chicken meat [14]. Kluytmans et al. showed a high degree of similarity of resistance genes and MLST patterns between the ESBL-E derived from meat and hospitalised patients [3]. Over-time, our data showed a decrease of CTX-M-1 like ESBL in comparison with the other ESBL types found. This could be due to the decrease in the use of intestinal antimicrobials in poultry (>60%), in our country during the same period [15]. However, more research about the trends in use and resistance is needed.

These periodic prevalence surveys are quality tools that give useful information about the local epidemiology of ESBL in a hospital and the surrounding community. This information is of major importance for the execution of good infection control policy [5]. The surveillance will be continued to obtain larger numbers and monitor further changes carriage rates, ESBL genes and nosocomial transmission, but at this stage we can conclude that the ESBL-E prevalence in our hospital was stable over time, which is remarkable since antimicrobial resistance increases worldwide. Furthermore, a minority of the detected ESBL-E were caused by nosocomial transmission, and a relative decrease of CTX-M-1 like ESBL genes was observed, possibly due to the decrease in the use of antibiotics in poultry.
